# Elevated Activin-A serum levels in patients with acute peripartum cardiomyopathy and during left ventricular recovery

**DOI:** 10.1093/eschf/xvag062

**Published:** 2026-02-23

**Authors:** Thomas Gausepohl, Tobias Jonathan Pfeffer, Tobias König, Dominik Berliner, Martina Kasten, Jason Roh, Denise Hilfiker-Kleiner, Johann Bauersachs, Melanie Ricke-Hoch

**Affiliations:** Department of Cardiology and Angiology, Hannover Medical School, Carl-Neuberg-Str., 30625 Hannover, Germany; Department of Cardiology and Angiology, Hannover Medical School, Carl-Neuberg-Str., 30625 Hannover, Germany; Department of Cardiology and Angiology, Hannover Medical School, Carl-Neuberg-Str., 30625 Hannover, Germany; Department of Cardiology and Angiology, Hannover Medical School, Carl-Neuberg-Str., 30625 Hannover, Germany; Department of Cardiology and Angiology, Hannover Medical School, Carl-Neuberg-Str., 30625 Hannover, Germany; Corrigan Minehan Heart Center, Massachusetts General Hospital, Harvard Medical School, Boston, MA, USA; Department of Cardiology and Angiology, Hannover Medical School, Carl-Neuberg-Str., 30625 Hannover, Germany; President, Hannover Medical School, Hannover, Germany; Department of Cardiology and Angiology, Hannover Medical School, Carl-Neuberg-Str., 30625 Hannover, Germany; Department of Cardiology and Angiology, Hannover Medical School, Carl-Neuberg-Str., 30625 Hannover, Germany

**Keywords:** Peripartum cardiomyopathy, Activin-A, Heart failure, Placental senescence, Left ventricular recovery, Plasminogen-activator-inhibitor 1

## Abstract

**Background and Aims:**

Peripartum cardiomyopathy (PPCM) is an idiopathic form of heart failure occurring in the peripartum phase. Elevated circulating levels of the senescence–associated–secretory–phenotype (SASP) factor Activin-A have been associated with heart failure severity in acute PPCM patients at baseline diagnosis. Here, we investigated Activin-A serum levels in the German PPCM registry in acute PPCM and during left ventricular (LV) recovery.

**Methods and results:**

Clinical data including LV ejection fraction (LVEF) and Activin-A serum levels were assessed at initial diagnosis (baseline [BL]) and during follow-up (FU) at 3 months (M) and 6M in PPCM patients from the German PPCM Registry (*n* = 151, mean age 33 ± 5 years) compared to postpartum healthy controls (*n* = 27, mean age 32 ± 5 years). Activin-A serum levels at BL were elevated (404 pg/ml; interquartile range [IQR]: 197–815, *n* = 151) compared to healthy postpartum controls (240 pg/ml, IQR:148–446, *n* = 27; *P* < .01) and remained persistently elevated above postpartum healthy controls at 3 M (418 pg/ml, IQR: 169–806, *n* = 100) and 6M-FU (520 pg/ml, IQR: 214–1131, *n* = 104). Activin-A levels at BL did not correlate with LVEF (Spearman *r*  *=* 0.10, *P* = .2416, *n* = 139), NT-proBNP (*r*  *=* 0.096, *P* = .2766, *n* = 131), CRP (*r*  *=* −0.0008, *P* = .9933; *n* = 110) or PPCM biomarker plasminogen-activator-inhibitor-1 (PAI-1) (*r*  *=* 0.095, *P* = .3273, *n* = 109). The majority of PPCM patients showed LV recovery 6M after initial diagnosis, indicated by improved LVEF (PPCM BL: 25%, IQR: 20–33, *n* = 152; 6M-FU: 52% IQR: 45–56, *n* = 128, *P* < .0001). Activin-A levels did not differ between full or incomplete LV recovery, or between patients with hypertensive pregnancy disorders.

**Conclusions:**

In PPCM patients from the German PPCM registry Activin-A serum levels were elevated at diagnosis, remained persistently high after 3M- and 6M-FU but were not associated with LV recovery.

## Introduction

Peripartum cardiomyopathy (PPCM) is an idiopathic form of acute systolic heart failure (HF) with peripartum onset.^1^ Left ventricular ejection fraction (LVEF) recovers frequently after acute PPCM.^2^ PPCM pathophysiology is likely driven by a multifactorial mechanism including the generation of the 16K-prolactin (16K-PRL), increased plasminogen–activator–inhibitor-1 (PAI-1), and soluble Fms-like tyrosine kinase-1 (sFlt1).^3,4^ Recently, Roh et al. reported an increase in circulating levels of senescence-associated–secretory–phenotype (SASP) proteins in PPCM patients with PAI-1 among the top 3 most upregulated proteins. Of these identified SASPs, Activin-A was also the most highly expressed in placentas from women with preeclampsia, a hypertensive disorder of pregnancy (HDP) strongly associated with PPCM.^5^ Increased serum Activin-A levels were confirmed in a cohort of PPCM patients recruited from the IPAC study, and were associated with metrics of HF severity, including BNP and NYHA.^6,7^ Inhibition of the Activin-A-receptor in mice with cardiomyocyte-specific knock-out of peroxisome-proliferator–activated-receptor-γ-coactivator­1α (PGC-1α), a common PPCM mouse model, had beneficial effects on cardiac systolic function.^7^

The aim of this study was to analyse Activin-A serum levels at diagnosis (baseline, BL) and after follow-up (FU) of 3 and 6 months (M), a time at which the majority of patients have already achieved partial to complete cardiac recovery, and to associate it to LV recovery and PAI-1 expression in the German PPCM registry.

## Methods

### Patients - data collection

The local ethics committee of Hannover Medical School approved this study, which conforms to the principles outlined in the Declaration of Helsinki. All patients provided written informed consent. Only patients who met with the PPCM diagnostic criteria of the European Society of Cardiology were included; the initial diagnosis is dated between January 2009 and November 2024.^1^ Assessments of demographic characteristics and clinical data were obtained from PPCM patients BL at diagnosis and at 6M-FU.

### Blood tests

Blood samples were collected in S-Monovette® tubes containing ethylenediaminetetraacetic acid (EDTA, plasma) or clot-activator (serum) BL (mean time postpartum 46 ± 55 days) and at the 3- and 6M-FU visits in PPCM patients and from healthy-postpartum women (PP-Ctrl) (22 ± 42 days postpartum, *P* < .005). Plasma or serum was separated by centrifugation at 1500 rpm for 10 min and stored at −80°C. PAI-1 plasma levels were measured using the Quantikine ELISA for human total serpin E1/PAI-1 Immunoassay (R&D systems, DTSE100)^8^, and Activin-A serum concentrations were determined using a commercial ELISA kit (R&D systems, DY338 and DY008B),^7^ both according to the manufacturer's protocol. Laboratory workup was performed as part of the routine analysis by hospital laboratories for *N*-terminal pro-brain natriuretic peptide (NT-proBNP) and C-reactive protein (CRP) at BL and 6M-FU.

### Statistical analyses

Statistical analysis was performed using GraphPad Prism version 10.0 for Mac OS X (GraphPad Software, San Diego, California, USA). The D’Agostino & Pearson normality test was used to test the data against the hypothesis of normal distribution. Continuous parametric data were analysed using an unpaired t-test. Non-parametric data of two groups were tested with Mann–Whitney *U* test, in case of multiple comparisons, the Kruskal–Wallis test with Dunn’s post-test was performed. A *P* value of <.05 was considered statistically significant. Analysis for correlation between two parameters was performed with Spearman r for nonparametric data.

## Results

### Clinical characteristics

The clinical characteristics of the PPCM patients at BL (*n* = 152), 6M-FU (*n* = 128), and PP-Ctrl (*n* = 27), including age, parity, CRP, and LVEF, are provided in *Table 1*. Cardiac function and clinical markers of HF improved during 6M-FU, as indicated by an increased LVEF as well as decreased NT-proBNP serum levels and New York Heart Association (NYHA) stages in PPCM patients (*Table 1*).

**Table 1:** Clinical characteristics of PPCM patients compared to PP-Ctrl

Parameters	PP-Ctrl (*n* = 27)	PPCM BL (*n* = 152)	PPCM 6M-FU (*n* = 128)
Age (years)	32 ± 5	33 ± 5	-
Gravida	1 (1–2)	2 (1–3)	-
Parity	1 (1–2)	2 (1–3)	-
Hypertensive pregnancy disorders	0 (0)	31 (20)**	-
Bromocriptine/cabergoline treatment	0 (0)	136 (89)****	-
Left ventricular ejection fraction (%)	64 (63–67)	25 (20–33)****	52 (45–56)***,^####^
NT-proBNP (ng/L)	—	2938 (858–6918)	128 (76–222)^####^
C-reactive protein (mg/L)	—	9 (5–35)	2 (1–5)^####^
New York Heart Association stage	—	3 (2–4)	1 (1–2)^####^

Data are presented as mean ± standard deviation (SD), median (interquartile range (IQR)), or *n* (%). **P < .01, ***P < .001, ****P < .0001 vs. PP-Ctrl, ^####^*P* < .0001 vs. PPCM BL, Mann–Whitney *U* test or to Kruskal–Wallis test with Dunn’s post-test for multiple comparisons.

### Activin-A serum levels in acute PPCM

**Figure 1:**
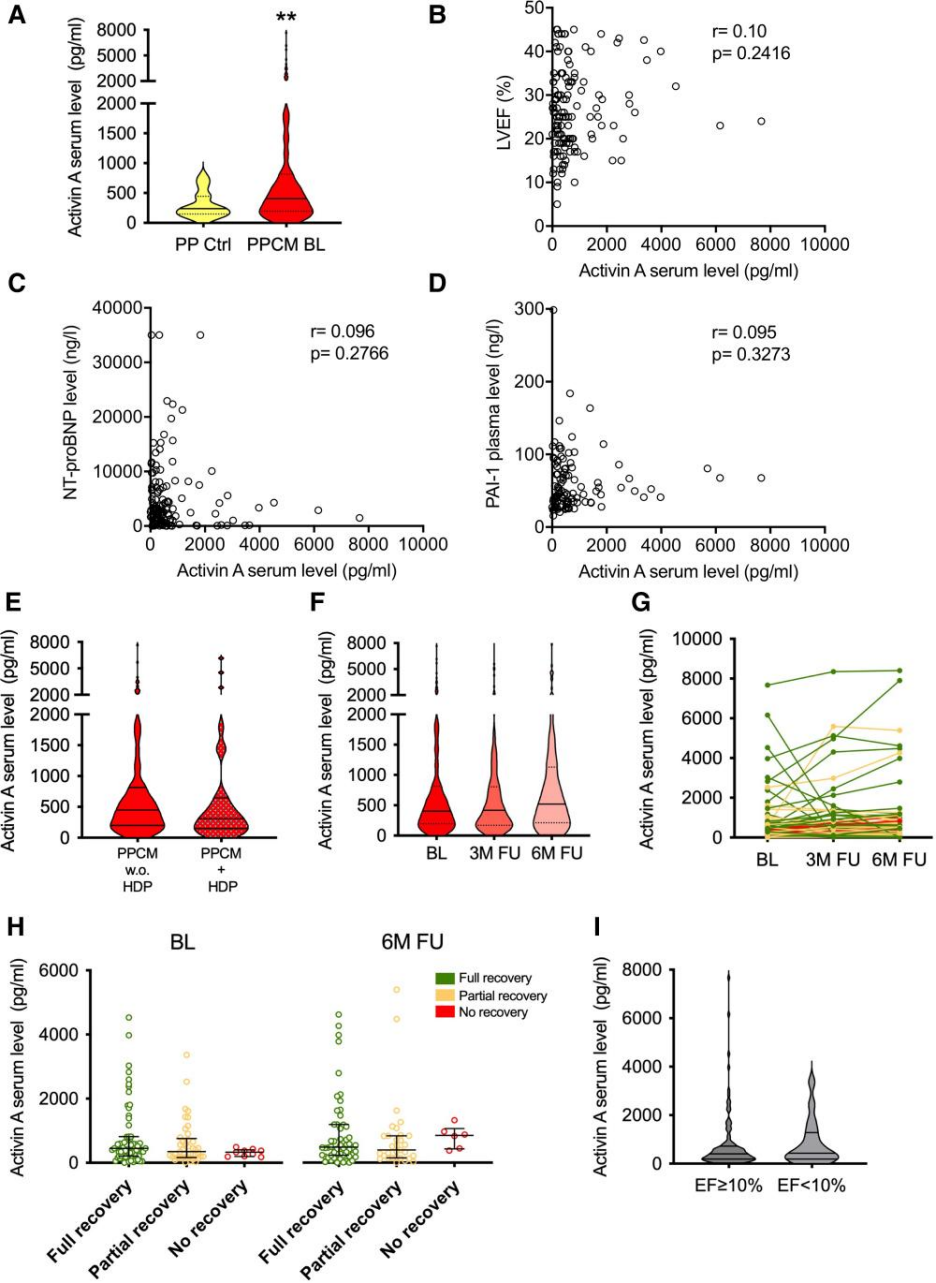
Increased Activin-A serum levels in PPCM patients BL, 3- and 6M-FU. (*A*) Activin-A serum levels of PP-Ctrl (*n* = 27) and PPCM (*n* = 151) patients at BL shown as violin plot. (*B–D*) Ozone correlation analysis of LVEF (*n* = 139), NT-pro-BNP serum level (*n* = 131), and PAI-1 plasma level (*n* = 109) with the Spearman coefficient of PPCM patients at BL with Activin-A serum level. (*E*) BL Activin-A serum levels of PPCM patients without (w.o.) hypertensive disorders of pregnancy (HDP) (*n* = 120) and PPCM patients with HDP (*n* = 30) shown as a violin plot. (*F*) Violin plot stating Activin-A serum levels of PPCM patients at BL (*n* = 151), 3M-FU (*n* = 100), and 6M-FU (*n* = 104). (*G*) Course of Activin-A serum levels in PPCM patients with at BL, 3- and 6M-FU, colours indicate LV recovery at 6M-FU (full-recovery *n* = 42, partial-recovery *n* = 28, no-recovery *n* = 6). (*H*) Scatter dot plot stating Activin-A serum levels of PPCM patients regarding their LV recovery at 6M after initial diagnosis at BL (full-recovery *n* = 71, partial-recovery *n* = 41, no-recovery *n* = 9) and 6M-FU (full-recovery *n* = 58, partial-recovery *n* = 31, no-recovery *n* = 6). (*I*) BL Activin-A serum levels of PPCM patients with LVEF improvement ≥10% (*n* = 104) versus those with <10% (*n* = 17) at 6M-FU compared to initial diagnosis shown as a violin plot. Data are presented as median with (interquartile range (IQR)), **P < .01, Mann–Whitney-*U* test. Two values at BL and two values at 6M-FU belonging to the full recovery cluster were outside the axis limits and could not be mapped due to the representability.

Activin-A serum levels of PPCM patients were elevated to a median of 404 pg/ml (IQR: 197–815) at BL compared to healthy PP-Ctrl (240 pg/ml, IQR: 148–446) (*Figure 1A*). However, LVEF, NT-proBNP, and CRP (r = −0.0008, *P* = .9933; *n* = 110) serum levels of PPCM patients at BL did not correlate with respective Activin-A serum levels (*Figure 1B, C*). Activin-A serum levels at BL also did not associate with PAI-1 plasma concentrations or concurrent HDP in PPCM patients (*Figure 1D* and *E*).

### Persistently high Activin-A serum levels in PPCM at 3- and 6M-FU

Measurement of Activin-A serum levels in PPCM patients at 3- and 6M-FU revealed persistently elevated Activin-A levels at these timepoints after diagnosis (BL: 404 pg/ml, IQR: 197–815; 3M-FU: 418 pg/ml, IQR: 169–806; 6M-FU: 520 pg/ml, IQR: 214–1131) (*Figure 1F, G*).

### Activin-A serum levels and LV recovery

Analysis of the cohort according to LV recovery status after 6M revealed that neither Activin-A serum levels at BL nor at 6M-FU differed significantly between patients with full LV recovery (6M-FU: LVEF > 49%), partial LV recovery (6M-FU: LVEF 36–49%) or no LV recovery (6M-FU: LVEF ≤ 35%) as shown in *Figure 1H*. Activin-A serum levels of PPCM patients whose LVEF improved ≥10% at 6M-FU also were not significantly different compared to PPCM patients with <10% LVEF improvement at 6M-FU (*Figure 1I*).

## Conclusion

This study confirmed increased circulating levels of the SASP factor Activin-A in a large cohort of acute PPCM patients from the prospective German PPCM registry as compared to healthy postpartum controls. However, surprisingly, Activin-A serum levels did not decline during 3- and 6M-FU, a time at which the majority of the patients already showed partial to complete cardiac recovery. Activin-A levels did not correlate with LVEF, NT-proBNP, CRP or PAI-1 concentrations in the German PPCM cohort, and did not associate with the degree of LV recovery or concurrent HDP (*Figure 1*). In contrast to the IPAC cohort,^9^ an association between BL Activin-A levels and BNP, NYHA class, or HDP could not be detected in the German cohort, which could be due to differences between the study groups, including differences in baseline demographics such as potential contributions from African genomic ancestry, inclusion criteria and timing of BL sample collection of PPCM patients, lower percentage of HDP present in the German PPCM patients and treatment with dopamine D2 agonists in the German cohort. Although elevated Activin-A levels in the peripartum phase are mostly derived from the placenta,^7^ the persistence of high Activin-A serum levels at 3- and 6M-FU suggests increased extraplacental Activin-A expression occurs in PPCM patients. Notably, most PPCM patients in the German PPCM registry received the dopamine agonist bromocriptine, leading to the inhibition of the 16K-PRL pathway.^10^ LV recovery in the presence of persistently elevated Activin-A serum levels suggests that Activin-A is not a major factor regulating cardiac function in the postpartum recovery phase of PPCM. Moreover, neither the prolactin pathway, nor the nursing hormone itself or its *N*-terminal 16K-PRL metabolite seems to modulate Activin-A levels. Notably, preeclampsia patients also exhibit persistently elevated Activin-A levels later in life that are associated with subclinical cardiac dysfunction.^11,12^ Activin-A is not only secreted by senescent cells, but is also a potent inducer of paracrine senescence.^13^ It is thus possible that the persistence of the SASP factor Activin-A may promote premature ageing of the cardiovascular system in women with preeclampsia or PPCM. This potential contribution to their increased risk of cardiovascular disease later in life needs to be investigated in further studies with large international patient cohorts and longer-term FU timepoints.

### Limitation

Although consistently elevated Activin-A levels are present in PPCM patients between BL and 6M-FU, the potential impact of the difference in timing of blood sample collection between PPCM patients and healthy-postpartum controls might have an influence on Activin-A levels.

## Declarations

### Data Availability

The data underlying this article will be shared reasonable request to the corresponding author.
